# Evidence of Anomalously Low δ^13^C of Marine Organic Matter in an Arctic Fjord

**DOI:** 10.1038/srep36192

**Published:** 2016-11-09

**Authors:** Vikash Kumar, Manish Tiwari, Siddhesh Nagoji, Shubham Tripathi

**Affiliations:** 1 National Centre for Antarctic & Ocean Research, Vasco-da-Gama, 403804, Goa, India

## Abstract

Accurate estimation of relative carbon deposition (marine vs. terrestrial) is required for understanding the global carbon budget, particularly in the Arctic region, which holds disproportionate importance with respect to global carbon cycling. Although the sedimentary organic matter (SOM) concentration and its isotopic composition are important tools for such calculations, uncertainties loom over estimates provided by organic-geochemical bulk parameters. We report carbon and nitrogen concentrations and isotopes (δ^13^C and δ^15^N) of SOM at an Arctic fjord namely Kongsfjorden. We find that the bound inorganic nitrogen (ammonium attached to the clay minerals) forms a significant proportion of total nitrogen concentration (~77% in the inner fjord to ~24% in the outer part). On removing the bound nitrogen, the C/N ratio shows that the SOM in the inner fjord is made up of terrestrial carbon while the outer fjord shows mixed marine-terrestrial signal. We further show that the marine organic matter is unusually more depleted in ^13^C (~−24‰) than the terrestrial organic matter (~−22.5‰). This particular finding also helps explain high δ^13^C values of SOM as noted by earlier studies in central Arctic sediments despite a high terrestrial contribution.

Although atmospheric carbon represents only a small part of the total carbon present on earth^1^,^2^, it’s ever increasing rate over the last few centuries is widely recognized as an existential threat to life on this planet. The atmosphere is essentially a large flux reservoir for carbon whose concentration depends on the exchanges with other reservoirs^3^. One such exchange is the burial of land and marine synthesized organic carbon as sedimentary organic matter (SOM) into the ocean and forms an important part of the global carbon cycle. Apart from serving as a tool for studying depositional environment, transport pathways, remineralization, and past climate conditions, source characterization of SOM is required to estimate the burial rates of marine and terrigenous organic matter^4^,^5^,^6^,^7^,^8^. Although various geochemical proxies such as the C/N ratio of SOM^6^,^9^, specific biomarkers^10^ and Rock-Eval parameters^11^ are used to identify relative composition of land and marine derived components of SOM, it’s carbon isotopic composition is one of the most widely used tracer^12^,^13^,^14^,^15^. However, gross differences exist between different estimates^16^ primarily on account of proxy specific limitations. δ^13^C based provenance determination is limited in its applicability due to uncertainty associated with land and marine end-member isotopic compositions. This problem is of particular interest in the Arctic region, given it’s uniqueness as compared to other world oceans and its disproportionate importance in the global carbon budget^17^ and global climate system at large.

Terrestrial carbon is an important source of organic carbon preserved in marine sediments. The fraction making its way into the ocean, having survived a great deal of oxidizing pressure during transport, is highly refractory and thus constitute a high proportion in buried sediments not only in the near shore environments but also in the open ocean sediments. It is also the one which may be subject to significant change on account of land-use changes due to anthropogenic activities^18^,^19^. Marine organic carbon, on the other hand, is highly labile and suffers significant degradation during its vertical transport. Less than 1% of marine carbon produced in sub-surface waters gets buried with sediments depending upon the type of depositional environment^20^. Although riverine and eolian flux (0.43–0.75 Gt/y) into the oceans is very small compared to the primary productivity (30–50 Gt/y), up to 0.1 Gt/y of the total 0.16 Gt/y of estimated burial flux may be from the terrestrial source alone^17^. The Arctic ocean plays an important role in the global carbon cycle by accounting for 7–11% of the global burial flux, although it represents only 2.5% of the world ocean area^17^. This is primarily due to high terrestrial deposition in central and marginal environments of Arctic Ocean where transportation is facilitated via sea ice^16^.

Assumptions regarding the isotopic composition of terrestrial and marine end members limit the applicability of bulk isotopic composition of SOM for source characterization (provenance). This is especially important in the Arctic region where uncertainty exists for marine end-member isotopic composition on account of high pCO_2_ in cold surface water. The δ^13^C values between −26‰ to −28‰ is often taken as the terrestrial end member for C-3 plants upon an estimated 20‰ fractionation from the atmospheric CO_2_ value of −7‰^5^,^6^,^21^,^22^. C-4 plants play a minor role in high-latitude regions like the Arctic Ocean^23^. In mid to low latitude regions, δ^13^C value of −20‰ to −22‰ is commonly assigned to marine organic matter^5^. However, a range of values between −16.7‰ to −30.4‰ has been suggested for high latitudes, making it difficult to have reliable assumptions regarding δ^13^C of marine organic carbon in the Arctic region^24^,^25^. Yet another problem with respect to organic matter source characterization in the Arctic sediments is the impact of land derived bound nitrogen on the C/N ratio of bulk sediments^8^. The presence of high clay content in Arctic sediments leads to high proportion of bound inorganic nitrogen^9^. If the inorganic component is not actively removed, the C/N ratio based source characterization may be significantly underestimated with respect to the terrestrial organic matter.

One of the ways to address the problem of constraining isotopic end-member composition is to investigate the spatial variability of geochemical properties of SOM along a steep productivity gradient with adequate terrestrial contribution. Productivity gradient leads to variation in the mixing ratio between the marine and the terrestrial carbon along the gradient. Isotopic measurements along the gradient would thus help in constraining the end-member isotopic compositions. In this study, we have investigated the spatial variability of SOM and its carbon and nitrogen isotopic composition using surface sediments at a site with steep marine productivity gradient and ample terrestrial input. The study region is Kongsfjorden (Fig. 1), a fjord system in the Arctic region, located in the western part of Spitsbergen Island, the largest among the Svalbard archipelago in the Arctic Ocean. It is known for its strong hydrological, sedimentological and biological gradients along the axis^26^. Previous records from the region^27^,^28^ are limited and a knowledge gap exists. Results from the spatial variability of SOM in surface sediments will also form the groundwork for high-resolution long core studies from the region.

## Study Area

Kongsfjorden is situated between 78°40′ and 77°30′N and 11°3′ and 13°6′E. Approximately 20 km long, its width varies between 4 km and 10 km. The inner fjord is marked by relatively shallow depth (less than 100 m) as compared to the outer fjord (~250 m). Around 80% of the catchment area is glaciated, with two active glaciers towards the head and three towards the northern coast. The southern coast is relatively ice free with no active glacier. Western Spitsbergen is flanked by a northerly warm West Spitsbergen current (WSC), with Atlantic water mass (AW) constituting the top 600 m. A cold coastal current with Arctic water (ArW) also traverses the shelf in the outer fjord region. The presence of these currents towards the mouth of the fjord give rise to fronts and instabilities associated with them causes exchange of warm saline shelf transformed Atlantic water with the cold freshwater from the glacial discharge. Mass balance studies from Kongsfjorden have shown that the major contribution of fresh water into the fjord comes from the glacial discharge^29^ since precipitation plays a limited role. Climate change can affect both cross-shelf exchange of water mass between Atlantic, Arctic and freshwater as well as glacial discharge into the fjord. The temperature of AW in the WSC has increased in the recent years owing to coupled ocean-atmosphere interaction associated with the positive phase of North Atlantic Oscillation (NAO)^30^.

## Results and Discussion

### Total organic carbon and total nitrogen concentration

**Figure 2:**
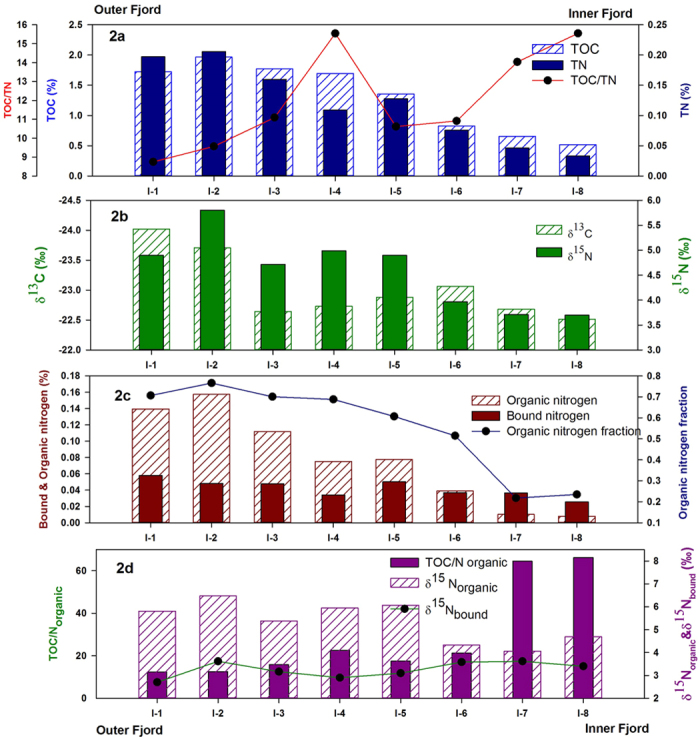
(**a**) Spatial variability of TOC, TN and TOC/TN of surface sediments along the fjord axis. Systematic increase in TOC and TN and decline in TOC/TN ratio is observed from inner fjord to outer fjord. (**b**) δ^13^C and δ^15^N of surface sediments. The inner fjord sediments are depleted in ^15^N and enriched in ^13^C compared to the outer part. (**c**) Relative proportion of bound-inorganic nitrogen concentration and organic nitrogen concentration in surface sediments. Inorganic nitrogen constitutes a significant proportion of total nitrogen concentration. The organic fraction increases systematically away from the fjord head. (**d**) TOC//N_organic_, δ^15^N bound & δ^15^N_organic_ of surface sediments. High TOC/N_organic_ in the inner fjord is suggestive of predominantly terrestrial organic matter deposition in surface sediments in the inner part of the fjord. A more mixed marine-terrestrial signature is seen in the outer fjord region.

**Figure 3:**
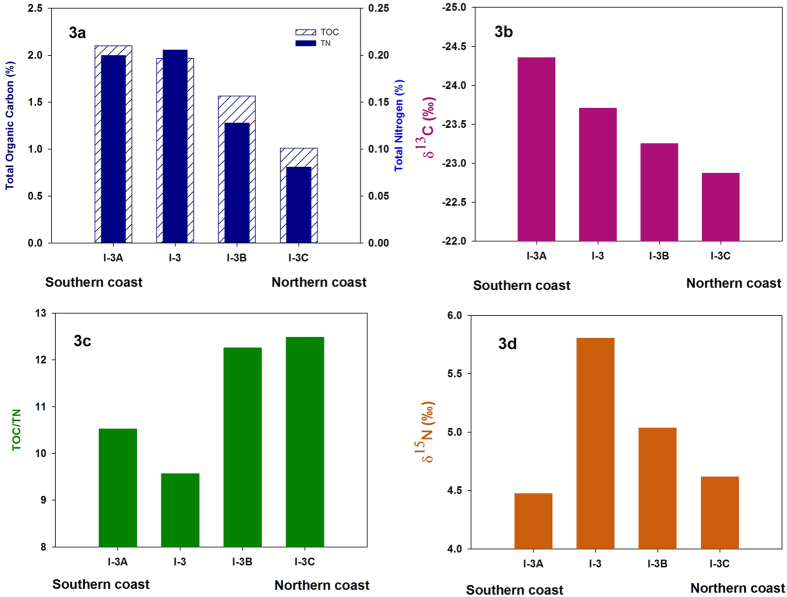
(**a**) TOC and TN, (**b**) δ^13^C, (**c**) TOC/TN and (**d**) δ^15^N variability across the fjord axis at station I-2. Note the similarity in gradient of SOM concentration and its carbon and nitrogen isotopic concentration along and across the fjord axis.

The organic carbon content in the surface sediments shows an increasing trend from approximately 0.5% at the fjord head to nearly 2% at the mouth. The total nitrogen concentration in surface sediments follows a similar trend with a minimum of 0.03% at the fjord head and a maximum of 0.21% in the outer part (Fig. 2a). Across the fjord axis, at I-2, TOC increases from nearly 1% at the glaciated northern coast to 2.1% close to southern coast. TN increases from 0.08% to nearly 0.2% along the same stretch (Fig. 3a). TOC/TN ratio in surface sediments (Fig. 2b) varies between 8 and 16. Spatial variability of TOC/TN in surface sediments shows a declining trend between the fjord head and the fjord mouth with the exception of station I-4 with abnormally high value. Across the fjord axis (Fig. 3c), it decreases between the northern and the southern coast at I-2.

TOC and TN in surface sediments at Kongsfjorden show a clear spatial gradient with lower values in the glacier dominated inner fjord and higher values towards the outer fjord. This trend is also preserved between the glaciated northern coast and glacier free southern coast, suggesting role of a common factor. Although high turbidity towards the fjord head, which give rise to a shallow photic zone, is often cited as the reason for such a trend in TOC and TN profile^27^, steep gradient in sedimentation rate and grain size sorting may have a role in producing such a profile. A high correlation between TOC and TN exist in the surface sediments (r^2^ = 0.88). The C/N ratio in marine organisms, given by Redfield^31^ and later modified^32^ ranges between 6.5 and 8.7 (C:N:P = 122 (±18):16:1). Terrestrial organic matter predominantly consists of compounds like cellulose, lignin etc. with a low nitrogen content. Therefore the C/N value in land derived organic matter is much higher and found to vary between 20 and 100^7^. TOC/TN in sediments from Kongsfjorden suggest mixed terrestrial-marine source with increasing marine influence towards the fjord mouth. Its decline across the fjord axis between the northern and the southern coast may be attributed to high terrestrial input at the northern coast. Abnormally high value at I-4 is probably due to shallow water depth and high current regime associated with low bathymetry. This is also evident from low microfossil abundance at I-4 dominated by *Cibicides Lobatulus* which is known for its association with high current conditions^33^ (manuscript under preparation). The effect of removing bound nitrogen on C/N ratio is discussed in a separate section.

### δ^13^C of sedimentary organic matter

Sedimentary organic matter in the inner fjord region is found to be more enriched in ^13^C compared to the outer fjord. The δ^13^C in surface sediments varies from nearly −22.5‰ in the inner fjord to about −24‰ in the outer fjord (Fig. 2b). The complete data is given in Table S1 in the Supplementary Information. Across the fjord axis, it varies from −24.35‰ at the southern coast to −22.87‰ at the northern coast close to station I-3 (Fig. 3b). Overall, the δ^13^C values in sediments are very close to that from other records from Svalbard region, but lower than those reported from the central Arctic region^9^.

**Figure 4:**
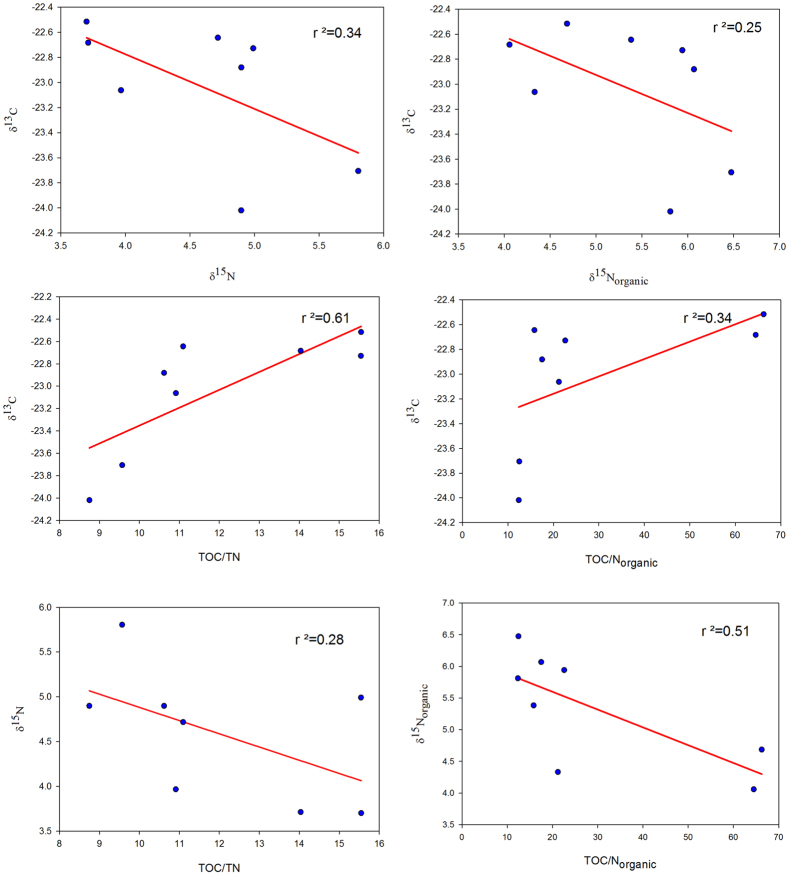
δ^15^N, δ^15^N_org_, TOC/TN and TOC/N_org_ versus each other and δ^13^C. Negative correlation between carbon and nitrogen isotopic composition of SOM and positive correlation between carbon isotopic composition and C/N ratio of SOM at Kongsfjorden is due to unusually more depleted marine organic matter.

**Figure 5:**
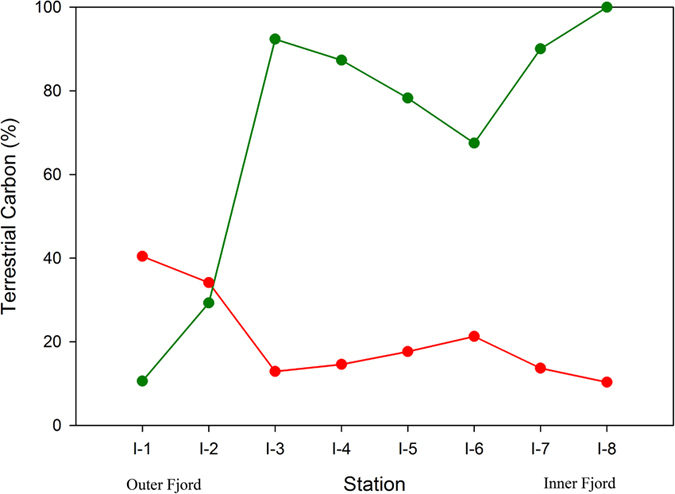
Terrestrial carbon concentration along the axis of the fjord. Red dots shows calculation based on fixed end-member δ^13^C values while green dots shows calculation based on new estimated end-member δ^13^C value (present study).

Variation of δ^13^C in sediments primarily reflects varying mixing ratio of terrestrial and marine organic carbon, with their distinct isotopic signatures. C-3 plants are the most common terrestrial source of organic matter in sediments with δ^13^C values ranging from −22‰ to −30‰ (average −27‰)^34^,^35^. δ^13^C of marine organic matter on the other hand is found to vary between −10‰ to −31‰, with most of them constrained between −17‰ and −22‰^36^. Low temperature regions with high pCO_2_ in surface waters leads to high fractionation during photosynthesis by plankton, resulting in more depleted carbon isotopic composition^37^. Trend shown by δ^13^C in surface sediments suggest terrestrial organic matter in Kongsfjorden is more enriched compared to that of the marine as the inner fjord region with predominantly terrestrial organic carbon (discussed under section “*Role of soil derived bound inorganic nitrogen concentration in surface sediments*”) has higher δ^13^C. Outer fjord region with increasing marine influence records lower δ^13^C. Similar explanation holds for δ^13^C trend across the fjord axis between the southern and the northern coast. Low δ^13^C of marine organic matter is also evident through a positive correlation between δ^13^C and the C/N ratio in surface sediments (Fig. 4). This result also seems to explain why the central Arctic region with high terrestrial input^38^,^39^ has more enriched carbon isotopic composition of SOM. An important implication of this finding is that fixed isotopic composition of end-members (lower ~−27‰ for terrestrial and higher ~−22‰ for marine) is often used for organic matter source characterization^9^, which in this case would result in an incorrect reconstruction. The difference between the estimates using above fixed end-member values and that inferred through the present study is shown in Fig. 5, for which the calculation is explained in the section “*Role of soil derived bound inorganic nitrogen concentration in surface sediments*”. δ^13^C vs. C/N plot shows mixed marine-terrestrial source for organic matter in surface sediment from Kongsfjorden (Supplementary Fig. S1). The effect of removing bound nitrogen on this plot is discussed under the section “*Role of soil derived bound inorganic nitrogen concentration in surface sediments*”.

### δ^15^N of sedimentary organic matter

δ^15^N in surface sediments shows an increasing trend between the inner fjord and the outer fjord. From around 3.7‰ at the head, it reaches to greater than 5‰ at the mouth. Across the fjord axis, 1.2‰ shift in δ^15^N can be seen at I-2 (Figs 2b and 3d). Overall δ^15^N in surface sediments are lower compared to central Arctic record^9^. δ^15^N trend in surface sediments, both along and across the fjord axis suggest high nutrient utilization in the glacier distal region (outer fjord) as compared to the inner region. Higher δ^15^N in outer fjord region indicates a low surface nitrate concentration. Nutrient laden Atlantic water influx in the outer fjord region may have offset some of the increase in δ^15^N with increasing marine productivity and may also explains the reason behind lighter nitrogen isotopic composition of SOM at Kongsfjorden as compared to the central Arctic record which has less Atlantic water influence. The correlation between δ^15^N and δ^13^C and that between δ^15^N and C/N ratio are both negative (Fig. 4). The negative correlation in our case is because a high δ^15^N is suggestive of a higher marine productivity while we have shown that both δ^13^C and the C/N ratio of marine productivity are low.

### Role of soil derived bound inorganic nitrogen concentration in surface sediments

**Figure 6:**
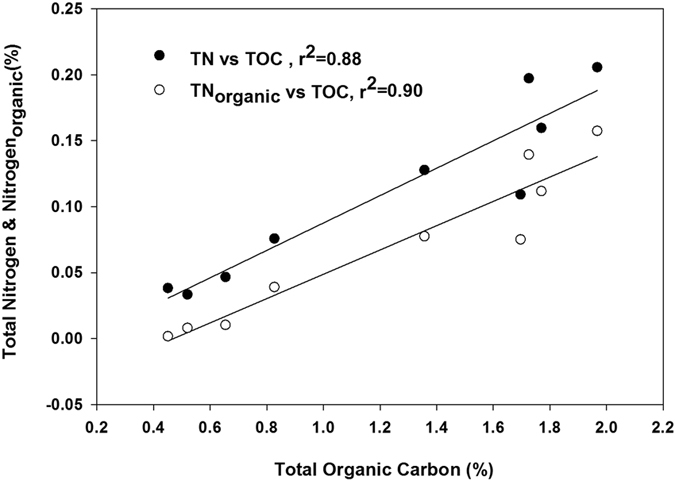
Correlation between TN and TOC and between N_organic_ and TOC. The difference in intercepts of the two regression lines is due to the presence of bound nitrogen in Sedimentary Organic matter.

High clay content in arctic sediments^39^,^40^ leads to significant amount of inorganic nitrogen bound to them^41^,^42^ as seen from the nitrogen content in KOBr-KOH treated surface sediments. The inorganic nitrogen bound as ammonium in clay minerals varies from about 0.03% in the inner fjord to nearly 0.06% in the outer fjord region in surface sediments, showing an increasing trend along the fjord axis (Fig. 2c). The organic nitrogen concentration (N_total_ − N_bound_) increases from 0.01% in the inner fjord to about 0.15% in the outer region. The organic fraction constitute between 24% to 76% of total nitrogen concentration, increasing sharply between the inner fjord and the outer fjord (Fig. 2c). The correlation coefficient between organic carbon and organic nitrogen improves slightly (r^2^ = 0.90) from that between TOC and total nitrogen in surface sediments (Fig. 6). C_organic_/N_organic_ ratio shows a more pronounced gradient between the inner fjord and the outer fjord as compared to that shown by C_organic_/N_total_ along the fjord axis. In the inner fjord it is greater that 60 indicating predominantly terrestrial source of SOM while dropping to around 20 in the central fjord and further down to 12 in the outer fjord suggesting increasing marine influence along the fjord axis (Fig. 2d). The δ^15^N_organic_, estimated assuming mass balance between organic and inorganic fractions, varies from 4.05‰ in the inner fjord to 6.47‰ in the outer fjord, significantly higher than δ^15^N_total_ at each location. These are lighter compared to organic nitrogen isotopic composition from central Arctic region^9^. δ^15^N_bound_ on the other hand exhibit a much narrower range (2.7‰ to 3.6‰) as compared to δ^15^N_organic_ with a mean of 3.28‰ (Fig. 2d).

Our record shows bound nitrogen constitute about 75% of total nitrogen in the inner fjord region and account for 25–30% in the outer fjord region. High clay content in Arctic sediments leads to significantly high amount of inorganic nitrogen attached to potassium rich minerals, coming from terrestrial runoff. A marked increase in bound nitrogen concentration from inner fjord to the mouth may have been from increasing clay content towards the open ocean. Interestingly, despite this increase in inorganic nitrogen along the fjord axis, the organic nitrogen fraction increases from as low as 23% to about 75% along the same stretch, indicating increasing marine influence in the glacier distal region. Overall organic nitrogen fraction as well as the total organic nitrogen content is higher than that reported from the central Arctic region^9^. The distinction between bound and organic nitrogen becomes more evident by comparing C/N ratio calculated from total nitrogen and organic nitrogen. The former significantly underestimates the terrestrial contribution to SOM; especially in the glacier proximal region with overwhelming terrestrial contribution (C/N_organic_ > 60) misrepresented as mixed marine-terrestrial (C/N_total_ ~15). This further lead us to attribute the δ^13^C signature coming out of the SOM from the inner most part of the fjord to be predominantly of terrestrial source, as can also be seen in the δ^13^C vs. C/N plot (Supplementary Fig. S1). This allows us to constrain the terrestrial end-member carbon isotopic composition at our site, which is unusually found to be more than the marine-endmember. Based on the TOC/N_organic_ values (assuming innermost value to be purely of terrestrial origin and a marine end-member value of 6) we estimated the δ^13^C of marine organic matter as −24.2‰. First we calculated the terrestrial fraction of SOM at station I-1 (eq. 1 below) using which δ^13^C_marine_ was calculated (eq. 2 below).
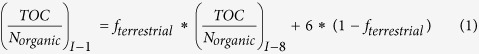

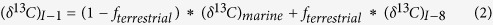
here, f_terrestrial_ represents terrestrial component of total SOM whereas subscripts represent respective station number (I-1, and I-8). Using this derived marine end-member δ^13^C value and the inferred terrestrial end-member δ^13^C value, terrestrial contribution to the SOM is obtained as per the equation shown below (eq. 3).
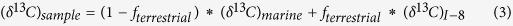


The difference between the terrestrial carbon concentrations based on fixed end-member δ^13^C values and those obtained using estimated end-member δ^13^C value is shown in Fig. 5.

Figure 6 shows that using N_organic_ improves the correlation between carbon and nitrogen and lowers the intercept of the linear regression curve between the two. A comparatively larger intercept shown by regression curve between TOC and TN indicates the presence of bound nitrogen. Narrow range exhibited by the isotopic composition of bound nitrogen suggests well-defined δ^15^N for inorganic nitrogen, possibly representing signal from land. Moreover lower values closer to atmospheric nitrogen (δ^15^N = 0‰) may have been from minor fractionation by land plants^43^. δ^15^N_organic_ shows a more pronounced spatial variability as compared to δ^15^N_total_ indicating better nutrient utilization in the outer fjord region. Lighter nitrogen isotopic composition compared to central Arctic record suggests the role of Atlantic water influx into the region.

## Summary

Multi-proxy record from surface sediments at Kongsfjorden shows a clear spatial gradient in bulk geochemical parameters, largely driven by the glacial-marine contrast. A similar gradient exists across the fjord axis between the glaciated northern coast and glacier free southern coast. Organic matter concentration increases with increasing distance from the glaciers. C/N ratio suggests SOM in the inner fjord region is dominated by terrestrial source while marine signature becomes dominant away from the glaciers. High clay content in sediments from Arctic region requires that bound nitrogen should be actively removed to interpret C/N and δ^15^N results. δ^13^C trend suggests marine organic matter to be more depleted in ^13^C compared to its terrestrial counterpart. This particular finding helps explain higher δ^13^C values in central Arctic sediments compared to Svalbard. It also has important implications for constraining end-member isotopic composition for δ^13^C based source characterization of sedimentary organic matter in Arctic region. δ^15^N shows better nutrient utilization away from the glacier and the influence of Atlantic water mass in western Spitsbergen.

## Methods

Eleven surface sediment grabs were collected at various water depths during August 2014 from Kongsfjorden (Table 1). Surface sediments were collected using Grab Sampler using the workboat “MS Teisten”. Samples are named according to their station number at each location. The samples were brought back to the laboratory (National Centre for Antarctic & Ocean Research, India) in frozen condition for further analysis.

The samples were freeze-dried before processing for elemental concentration (%C and %N) and isotopic analysis (δ^13^C and δ^15^N) of the sedimentary organic matter (SOM). A portion of the freeze-dried samples were finely ground for homogenization and further sub-sampled into two batches- (i) 2N HCl treated for Total Organic Carbon (TOC) measurement and (ii) untreated for determination of total nitrogen content. Additionally KOBr-KOH treatment was carried out on surface sediments for investigating the role of bound nitrogen present in the deposited sediments. HCl treatment removed carbonate from the sample for TOC and *δ*^13^C measurement. 20 ml of 2N HCl solution was added to 500–1000 mg of finely ground sediment. The mixture was swirled and allowed to stand overnight. The sample was then washed with double-distilled water and approximately 5 mg of treated sample was used for TOC and *δ*^13^C analysis^44^. KOBr-KOH treatment oxidizes organic nitrogen fraction, leaving behind bound nitrogen^9^,^45^. 6 ml of liquid bromine was added to ice-cooled 2M KOH at 0.5 ml/minute to prepare KOBr-KOH solution. 20 ml of this solution was added to 500–1000 mg of finely ground sediment. 60 ml of double distilled water was added and the mixture was boiled vigourously. After overnight cooling, the sample was washed with 0.5 M KCl to remove released ammonium. Presence of high concentration of potassium throughout the procedure insured that the released ammonium produced during oxidation doesnot bind with the clay present in the sample. The sample was again washed with double-distilled water. Approximately, 180 mg of KOBr-KOH treated sample was used for analysis. For Total Nitrogen concentration (TN) and *δ*^15^N measurement, approximately 50 mg of bulk ground sediment was used. Sediments from arctic region have been shown to contain significant amount of bound nitrogen of terrestrial origin^9^ which gets into the pool by replacing potassium in potassium rich clay minerals^46^, found in abundance in higher latitudes marine sediments. The organic nitrogen content is obtained by subtracting bound nitrogen content from the total nitrogen. *δ*^15^N_organic_ is obtained using isotopic mass balance (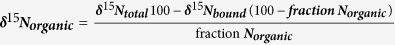). The analysis for elemental concentration and isotopic composition was carried out at the Marine Stable Isotope Laboratory (MASTIL) of National Centre for Antarctic and Ocean Research, Goa, India using an Isoprime Isotope Ratio Mass Spectrometer in continuous flow mode and coupled to an Elemental Analyser (Isoprime-Vario Isotope Cube) The precision of the organic carbon and nitrogen determinations was 0.31% and 0.24% respectively based on Sulfanilamide as the standard while that of carbon and nitrogen isotopic composition was 0.05‰ and 0.12‰ respectively (1σ standard deviation) based on cellulose (IAEA-CH-3) and ammonium sulphate (IAEA-N-1) as standards.

## Additional Information

**How to cite this article**: Kumar, V. *et al.* Evidence of Anomalously Low δ^13^C of Marine Organic Matter in an Arctic Fjord. *Sci. Rep.* **6**, 36192; doi: 10.1038/srep36192 (2016).

**Publisher’s note**: Springer Nature remains neutral with regard to jurisdictional claims in published maps and institutional affiliations.

## Supplementary Material

Supplementary Information

## Figures and Tables

**Figure 1:**
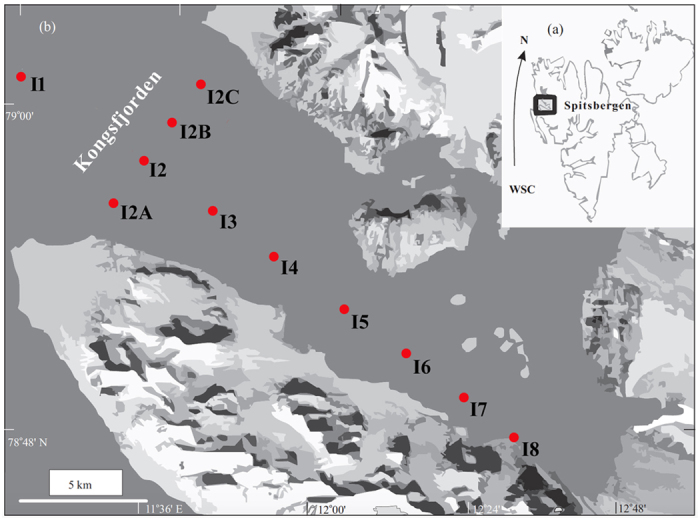
Map of Kongsfjorden and Spitsbergen (modified after Shetye *et al.* ^27^, with permission from John Wiley and Sons Inc.). Sampling locations are shown in red. Surface samples were collected along the fjord axis (I-1 to I-8) and across the fjord axis (I-2A to I-2C). Map modified using Adobe Photoshop CS5 ( www.adobe.com/in/products/photoshop).

**Table 1:** Sampling depth and location.

Sample	Depth	Lattitude (°N)	Longitude (°E)
I-1	250 m	79.0354	11.2836
I-2A	320 m	78.9844	11.5086
I-2	254 m	78.9931	11.5547
I-2B	230 m	79.0167	11.6250
I-2C	70 m	79.0414	11.7018
I-3	294 m	78.9755	11.6915
I-4	180 m	78.9587	11.8224
I-5	302 m	78.9408	11.9576
I-6	145 m	78.9228	12.0937
I-7	74 m	78.9931	12.3000
I-8	45 m	78.8951	12.3201
