# Supplementary material for: Evidence of Anomalously Low δ13C of Marine Organic Matter in an Arctic Fjord
Source: Sci Rep. 2016 Nov 9;6:36192. doi: 10.1038/srep36192 (PMC5101485; doi:10.1038/srep36192)
Supplement: Supplementary Information [file srep36192-s1.pdf]

Supplementary Information

Evidence of Anomalously Low $\delta^{13}C$ of Marine Organic Matter in an Arctic Fjord

Vikash Kumar*, Manish Tiwari, Siddhesh Nagoji & Shubham Tripathi

National Centre for Antarctic & Ocean Research, Vasco-da-Gama-403804, Goa, India

- Corresponding author: *vikash@ncaor.gov.in*; +(91)832-2525646

Table S-1. Sedimentary organic matter concentration (TOC, TN, $TN_{organic}$) and its carbon and nitrogen isotopic composition ($\delta^{13}C$, $\delta^{15}N$, $\delta^{15}N_{organic}$, $\delta^{15}N_{bound}$) of surface sediments at Kongsfjorden.

Sample ID	TOC (%)	TN (%)	$\delta^{13}C$ (‰)	$\delta^{15}N$ (‰)	TOC/TN	$N_{organic}$ (%)	TOC/$N_{organic}$	$\delta^{15}N_{organic}$ (‰)	$\delta^{15}N_{bound}$ (‰)
I-1	1.73	0.20	-24.02	4.90	8.75	0.139	12.38	5.81	2.70
I-2	1.97	0.21	-23.71	5.80	9.57	0.157	12.50	6.47	3.61
I-3	1.77	0.16	-22.64	4.72	11.09	0.112	15.84	5.38	3.16
I-4	1.70	0.11	-22.73	4.99	15.54	0.075	22.60	5.94	2.90
I-5	1.36	0.13	-22.88	4.90	10.62	0.078	17.51	6.07	3.09
I-6	0.83	0.08	-23.06	3.97	10.91	0.039	21.21	4.33	3.58
I-7	0.65	0.05	-22.68	3.71	14.04	0.010	64.50	4.06	3.62
I-8	0.52	0.03	-22.52	3.70	15.55	0.008	66.24	4.68	3.40
I-2A	2.10	0.20	-24.36	4.47	10.52				
I-2B	1.57	0.13	-23.25	5.04	12.26				
I-2C	1.01	0.08	-22.87	4.61	12.49				

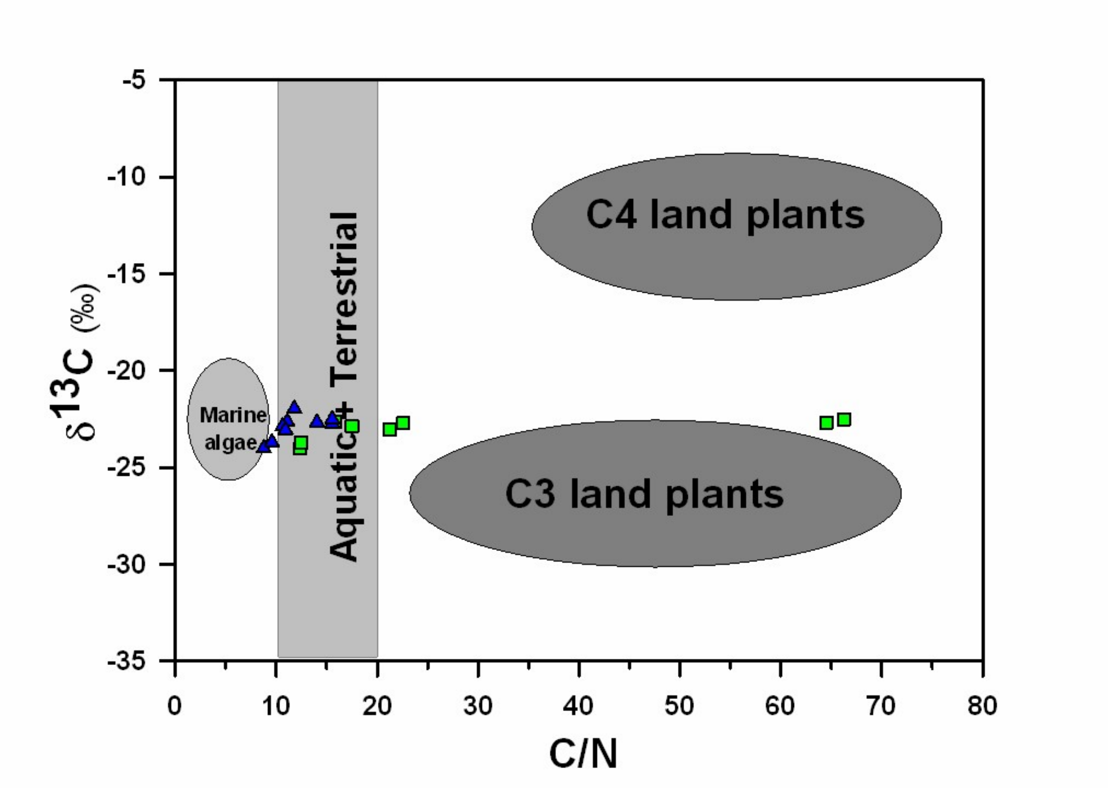

Figure S1. $\delta^{13}C$ vs C/N plot for surface sediments collected along the fjord axis (modified after Meyers, 1997[1]). TOC/TN values are shown as blue triangles while TOC/$N_{organic}$ values are shown as green squares.

References for SI

1. Meyers, P. A. Organic geochemical proxies of paleoceanographic , paleolimnologic , and paleoclimatic processes. *Org. Geochem.* **27,** 213–250 (1997).
